# Patient safety discourse in a pandemic: a Twitter hashtag analysis study on #PatientSafety

**DOI:** 10.3389/fpubh.2023.1268730

**Published:** 2023-11-16

**Authors:** Olena Litvinova, Farhan Bin Matin, Maima Matin, Bogumila Zima-Kulisiewicz, Cyprian Tomasik, Bodrun Naher Siddiquea, Jivko Stoyanov, Atanas G. Atanasov, Harald Willschke

**Affiliations:** ^1^Department of Management and Quality Assurance in Pharmacy, National University of Pharmacy of the Ministry of Health of Ukraine, Kharkiv, Ukraine; ^2^Ludwig Boltzmann Institute Digital Health and Patient Safety, Medical University of Vienna, Vienna, Austria; ^3^Department of Pharmacy, East West University, Aftabnagar, Dhaka, Bangladesh; ^4^Institute of Genetics and Animal Biotechnology of the Polish Academy of Sciences, Jastrzebiec, Poland; ^5^Department of Epidemiology and Preventive Medicine, School of Public Health and Preventive Medicine, Monash University, Melbourne, VIC, Australia; ^6^Swiss Paraplegic Research, Nottwil, Switzerland; ^7^Department of Anaesthesia, Intensive Care Medicine and Pain Medicine, Medical University Vienna, Vienna, Austria

**Keywords:** patient safety, Twitter, hashtag, COVID-19, adverse events, pharmacovigilance, vaccine, healthcare

## Abstract

**Background:**

The digitalization of medicine is becoming a transformative force in modern healthcare systems. This study aims to investigate discussions regarding patient safety, as well as summarize perceived approaches to mitigating risks of adverse events expressed through the #PatientSafety Twitter hashtag during the COVID-19 pandemic.

**Methods:**

This research is grounded in the analysis of data extracted from Twitter under the hashtag #PatientSafety between December 1, 2019 and February 1, 2023. Symplur Signals, which represents a tool offering a method to monitor tweets containing hashtags registered with the Symplur Healthcare Hashtag Project, was used for analyzing the tweets shared in the study period. For text analytics of the relevant data, we further used the word cloud generator MonkeyLearn, and VOSviewer.

**Results:**

The analysis encompasses 358′809 tweets that were shared by 90′079 Twitter users, generating a total of 1′183’384′757 impressions. Physicians contributed to 18.65% of all tweets, followed by other healthcare professionals (14.31%), and health-focused individuals (10.91%). Geographically, more than a third of tweets (60.90%) were published in the United States. Canada and India followed in second and third positions, respectively. Blocks of trending terms of greater interest to the global Twitter community within the hashtag #PatientSafety were determined to be: “Patient,” “Practical doctors,” and “Health Care Safety Management.” The findings demonstrate the engagement of the Twitter community with COVID-19 and problems related to the training, experience of doctors and patients during a pandemic, communication, the vaccine safety and effectiveness, and potential use of off-label drugs. Noteworthy, in the field of pharmacovigilance, Twitter has the possibility of identifying adverse reactions associated with the use of drugs, including vaccines. The issue of medical errors has been also discussed by Twitter users using the hashtag #PatientSafety.

**Conclusion:**

It is clear that various stakeholders, including students, medical practitioners, health organizations, pharmaceutical companies, and regulatory bodies, leverage Twitter to rapidly exchange medical information, data on the disease symptoms, and the drug effects. Consequently, there is a need to further integrate Twitter-derived data into the operational routines of healthcare organizations.

## 1. Introduction

The World Health Organization (WHO) identifies patient safety as one of the top concerns for global health. Currently WHO estimates that annually, sub-standard health care practices result in the deaths of 2.6 million people worldwide, disproportionally more in low- and middle-income countries. Globally, 4 out of 10 patients in primary and outpatient care experience adverse events, of which alarming 80% are preventable. The objective of patient safety, therefore is the mitigation of harm to patients during healthcare provision (1).

Unsafe medical practices encompass a range of issues, from errors in the prescription of medicines (e.g., in relation to dosing, timing of application, or drug interactions), with particular relevance to older adults, to nosocomial infections and non-compliance with safety rules during surgical procedures, injections, and blood transfusions. Furthermore, issues such as diagnostical errors, the use of radiation techniques, sepsis, and venous thromboembolism also contribute to the problem. These insights have been supported by multiple studies (2–9).

Extant literature shows that majority of adverse events associated with the provision of medical care are reported within the inpatient settings. More than half of these events in the hospital happen in the operating room and a third in the patient wards. Outside the hospital, general practitioners offices and patient’s homes are the most commonly implicated places of patient harm (10, 11).

Today, the digitalization of healthcare has great potential to improve the quality of medical care, taking patient safety into account. The professional utilization of social networks, including the healthcare field, is an area of constant growth. Twitter has emerged as instrumental in global public sphere research, as it allows rapid dissemination of information across borders and languages. Internet discussions tagged with hashtags, and the social groups engaged in these conversations, are shaping an interdisciplinary field that intertwines public opinion study, public sphere research, social network analysis, and conference outcomes (12–14). Thus, Twitter can be an additional source of information with relevance for patient safety. In this context, the Digital Health and Patient Safety Platform (DHPSP), an open innovation hub, was developed to facilitate collaborative research and development of innovative digital products and personalized solutions focused on enhancing patient safety and human health, and Twitter communication has been instrumental tool for the development of this platform. Along this line, it has been determined that COVID-19, health care, digital health technology, and scientific communication were the top subjects that caught the attention of the #DHPSP Twitter community (15).

Within another relevant research example, Nakhasi et al. carried out an analysis of tweets related to medical errors. Out of 1.006 tweets analyzed, 839 (83%) reported different error types: procedural (26%), medication (23%), diagnostic (23%), and surgical (14%). The authors highlight the potential of Twitter for patient safety-related analysis (16). Another study by Sharma et al. highlights that patients use Twitter to share personal, often disturbing, healthcare experiences (17).

A study by Chai et al. on the effectiveness of using the medical toxicology tweetchat (#firesidetox) collected growing number of impressions, and tweets led the authors to conclude that the tweet-chat model is feasible and well-received for discussing relevant medical toxicological topics (18).

Twitter scientometric analysis is also increasingly employed to evaluate the research outcomes related to patient safety. The data obtained from this resource is a rich source of information for analysis.

Although the WHO has declared the end of COVID-19 pandemic as a global health emergency, there is a threat of new variants of the virus. Unfortunately, the number of global COVID deaths recorded by WHO has reached 7 million over the 3 years, yet the director-general of the World Health Organization estimates that the true death toll is closer to 20 million (19). One key factor for the high mortality is the lack of initial experience in patient safety in the pandemic context, another - insufficient availability of vaccines. Lack of hospital preparedness and infrastructure (especially in low- and middle-income countries) and vaccine hesitancy have also been important contributors to negative public health outcomes.

The increased number of publications studying various aspects of patient safety, especially in light of COVID-19 pandemic, as well as the significant influence of Twitter on the development of all areas of society, have determined the relevance of studying the problem of patient safety based on the Twitter platform (20).

This study’s aim was to discern public discourse on patient safety and summarize perceived approaches to reducing the risks of adverse events using the hashtag #PatientSafety during the COVID-19 pandemic on Twitter.

## 2. Materials and methods

Tweets containing the hashtag #PatientSafety were analyzed from December 1, 2019, to February 1, 2023. The choice of such an interval is connected to the beginning of the COVID-19 pandemic in China. The study end date is related to a significant decrease in confirmed cases of COVID-19, according to WHO, in January 2023.

One of the features of Twitter is the conciseness of the tweets (messages) transmitted with it and the ability to instantly spread information along the chain. A hashtag is a type of tag marking with a starting sign #, a word or phrase used to identify digital content, such as a blog or post, corresponding to a specific category or subject (21). Users label tweets with hashtags to separate the themes of tweets and use them as means of finding or marking specific posts. The hashtag allows users to form an information wave consisting of messages on a specific topic, potentially forming a trend. The user can join the topic discussion at any stage by creating a response message.

We used the Symplur Signals tool, which allows researchers to track tweets containing hashtags registered in the Symplur Healthcare Hashtag Project (22). Symplur combines social media analytics and healthcare data to help users understand trending healthcare topics. When using a medical hashtag, this tool allow to generate an array of data regarding co-occurring hashtags, trending terms, the geography of participants, the influence of accounts, the dynamics of tweet activity, the Healthcare Social Graph Score, etc. The empirical basis of the study was an array of data from the social network Twitter on the hashtag #PatientSafety. The Symplur algorithm is used to measure the impact of specific accounts and the importance of the content they share for selected datasets and parameters. All tweets containing the hashtag #PatientSafety have been analyzed using Symplur signals without any restrictions on language, user location, or other parameters.

During the study, the most active stakeholders on Twitter were identified. In addition, the resulting data array was analyzed regarding the geography of the participants.

For text analytics of an array of trending terms obtained using the Symplur Signals tool, at the first stage we used the MonkeyLearn word cloud generator platform (available at https://monkeylearn.com/wordcloud/). Based on the analysis of trending terms, a visualization of significant terms (a word cloud) was built according to the frequency of their use. The color of words in the cloud depends on the frequency of the word in the text. Next, we identified three main directions based on the graphical representation of significant words. For each direction, the most frequently occurring trending terms are highlighted.

To identify the main topics of discussion with relevance to the hashtag #PatientSafety, an additional bibliometric analysis was carried out in the Web of Science database for the period from 2019 to 2023. The search for articles was carried out using the fields “title,” “abstract” and “key words.” The keywords used were “patient safety” and “Twitter.” The number of articles in this collection was 54. The array of data obtained was analyzed using the VOSviewer program version 1.6.18, which is in the public domain.

The Symplur Signals tool also generated a dataset for the hashtag #PatientSafety, containing information about co-occurring hashtags and their frequency. The paper presents the most frequently occurring related hashtags.

The Healthcare Social Graph Score was used as a quantitative assessment of the level of impact of the studied profiles. The value of this algorithm relates to the accurate identification and ranking of genuine influencers in any conversation about healthcare. Previous work by Mobarak et al. demonstrated the importance of this tool (23). It was revealed that the assessment of the social graph of health in scientific journals in the field of surgery positively correlates with their impact factor (the latter being more establish indicator of academic influence). A higher impact factor is associated with presence and activity on social media, particularly Twitter.

By monitoring more than 35,000 healthcare-related issues on Twitter in real-time, the Healthcare Social Graph Score is calculated. For each of the 35,000 topics, a top impact profile list for the previous year is prepared based on the tracking. Utilizing SymplurRank, an impact algorithm, rankings are produced. Symplur measures the caliber of the talks for each topic each week in addition to these rankings. The conversation volume and quality scores are combined to create a weighted estimate for the impact scores. The final step is to normalize each social media profile’s 52 weekly rankings and quality scores into a single value, which is then scaled from 0 to 100. The Healthcare Social Graph Score is the name given to this last figure (24).

To identify risks and develop approaches to their minimization, general scientific methods were used, in particular system, content analysis, scientific synthesis, logical generalization, and comparative analysis, among others.

This study does not contain any information about specific Twitter user accounts, and all the data presented is anonymous.

## 3. Results

The analysis yielded 358′809 tweets, shared by 90′079 Twitter users, amassing a total of 1′183’384′757 impressions (views).

**Figure 1:**
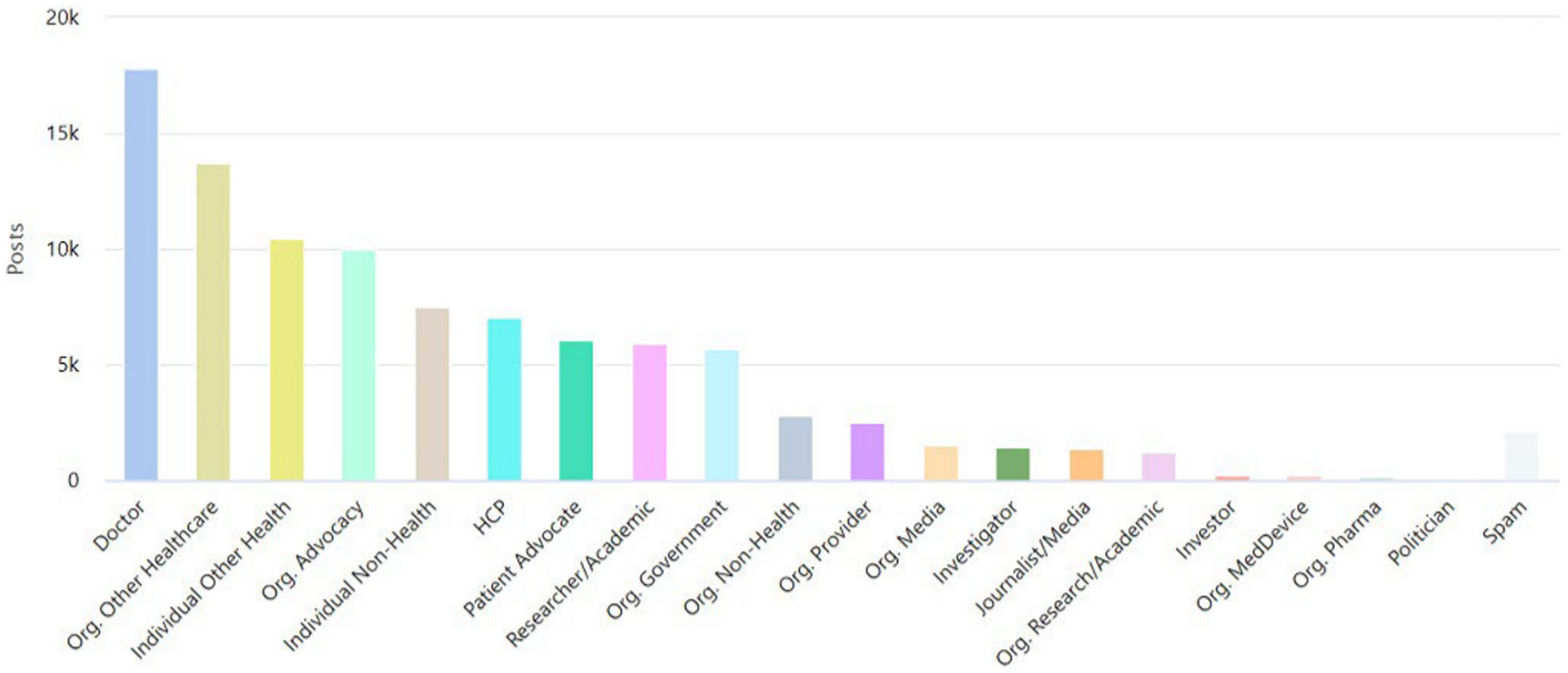
The number of tweets related to the hashtag #PatientSafety shared by major health care stakeholders.

Studies of the composition of stakeholders using the hashtag #PatientSafety unveiled heterogeneous groups, with overlapping activities related to the field of healthcare. Figure 1 showcases the activity associated with the #PatientSafety hashtag use on Twitter by these specific groups.

Symplur categorizes healthcare stakeholder accounts as follows^1^: doctor: those believed to be licensed, MDs, DOs, and PhDs who bill directly for services. Also includes medical residents; HCP: those believed to be other healthcare professionals (i.e., nurses, dietitians, respiratory therapists, nurses, pharmacists, etc.); patient advocate: person who publicly self-identify in their Twitter bio as a patient advocate for a specific disease or condition; caregiver: a professional caregiver or a person who is currently or has been a caregiver of a family member or other closely associated individual; researcher/academic: person who is working in the field of health-related research and/or academia (a PhD who does not treat patients falls in this category); journalist/media: person whose profession is journalism or other news-related media. Doctors who are editors of journals do not get this label; individual other health: person working in the healthcare industry in a nonclinical role; individual non-health: person not known to be directly working in the healthcare industry; org. Provider: inpatient facilities, medical groups, labs, imaging centers, and other outpatient facilities; org. Research/academic: accredited schools of higher learning (i.e., universities, colleges, etc.) and healthcare research institutions/centers; org. Advocacy: an organization focused on a specific set of health issues or medical specialty for the purpose of support, guidance, and education; org. Media: all organizations whose primary purpose is publishing or broadcasting; org. Other healthcare: organizations fulfilling roles within the healthcare industry but not providing direct clinical care; org. Non-health: all organizations not falling into an established category.

The data indicates that doctors (18.65% of all tweets), organizations in healthcare (14.31% of all tweets), and individuals in other health-related fields (10.91% of all tweets) held the top three places of stakeholder ranking.

**Figure 2:**
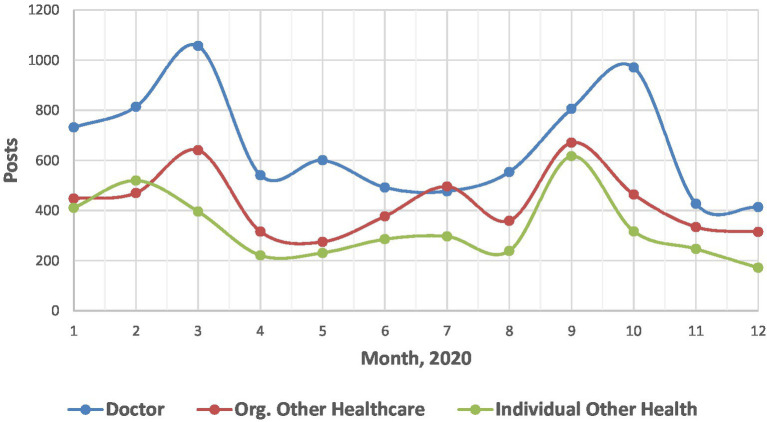
Dynamics of publication activity associated with the hashtag #PatientSafety by leading stakeholders on Twitter (2020).

With WHO declaration of the coronavirus pandemic in March 2020, the life of society has moved to the online space. The tweet-posting activity of leading stakeholders in the selected study area had a pronounced positive trend in March 2020 (Figure 2). The second wave in the fall is associated with Pfizer’s discussion of vaccine development.

**Figure 3:**
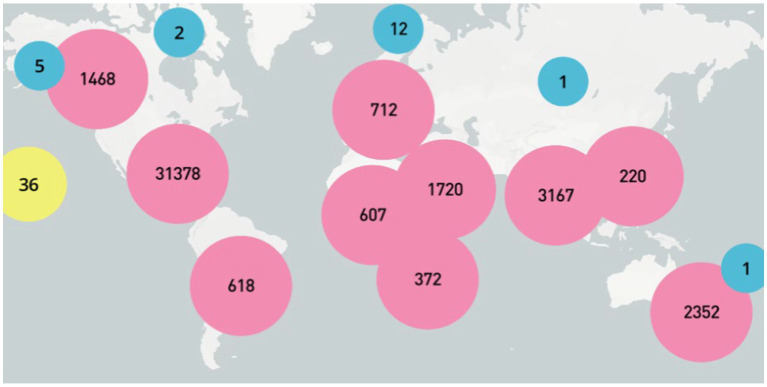
Regional distribution of the posted tweets.

Geographical analysis of tweets (Table 1), including the hashtag #PatientSafety, revealed that more than a third of tweets (60.90%) were published in the United States. Canada and India followed in the second and third positions, respectively. More than 1% of tweets were posted by each Australia, Saudi Arabia, Switzerland, and Nigeria. In total, more than 100 countries were found to have made at least one tweet post dedicated to patient safety. A map (Figure 3) has been constructed that clearly illustrates that interest in the research subject exists worldwide.

**Table 1:** Top 5 countries sharing #PatientSafety tweets.

	Countries	Users	Percentage (%)
1.	United States	25,985	60.90
2.	Canada	6,095	14.28
3.	India	2,543	5.96
4.	Australia	2,164	5.07
5.	Saudi Arabia	497	1.17

One of the interesting indicators of Twitter is the analysis of co-occurring hashtags with #PatientSafety. Users, in conjunction with the hashtag #PatientSafety, most frequently used the following hashtags: #Healthcare, #COVID19, #patientcare, etc. (Table 2).

**Table 2:** Co-occurring hashtags with #PatientSafety.

*N*	Hashtags	Count
1.	#Healthcare	23,210
2.	#COVID19	11,213
3.	#patientcare	9,501
4.	#worldpatientsafetyday	8,807
5.	#hospital	8,222
6.	#digitalhealth	7,403
7.	#doctors	6,934
8.	#MedTwitter	6,649
9.	#MedEd	6,413
10.	#health	6,112
11.	#HealthTech	6,087
12.	#Quality	5,828
13.	#AHRQ	5,171
14.	#medicine	5,099
15.	#nurse	5,052
16.	#NHS	4,646
17.	#patients	4,580
18.	#patientexperience	4,096
19.	#ptsafety	4,070
20.	#publichealth	3,717

In order to identify the most influential accounts posting tweets using #PatientSafety, an analysis was carried out following the SymplurRank algorithm. Among the top influencers, there was an equal proportion of males and females, comprising 32% of each. These accounts included those of healthcare executives, doctors, researchers, nurses, editors, etc. Organizations, institutions, and communities represented the remaining 36 percent of accounts.

**Figure 4:**
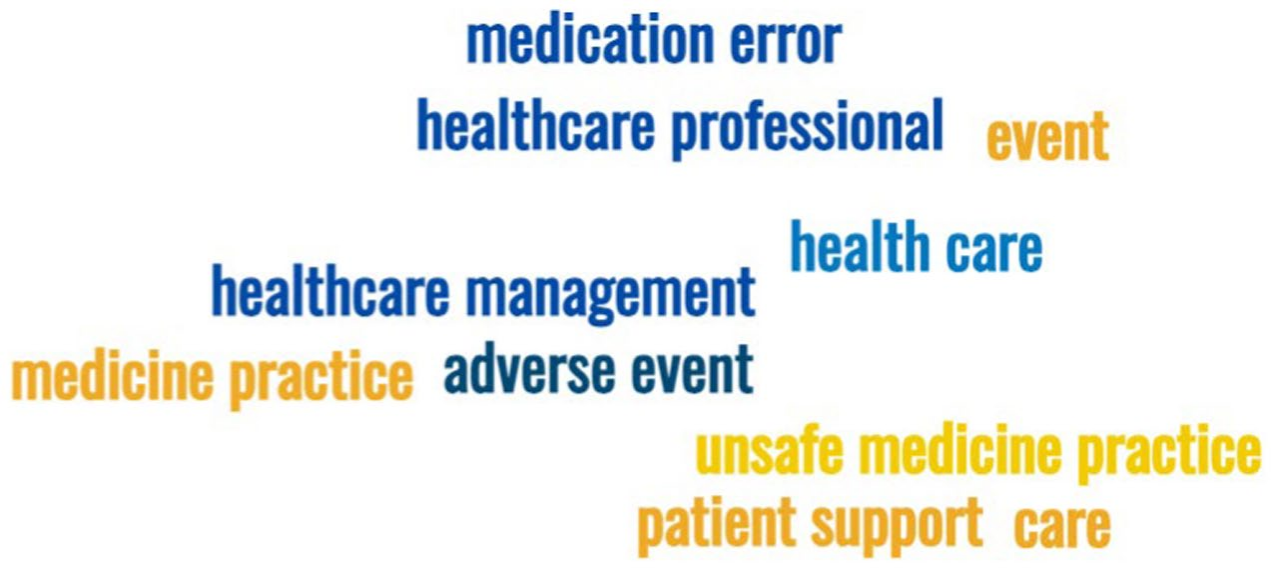
Visualization of significant trending terms (a word cloud) with the hashtag #PatientSafety by frequency of their use.

The thematic focus of tweets was also studied using the content analysis of trending terms using the MonkeyLearn word cloud generator platform. All terms were excluded from common words that have no value in the framework of this work, as well as words and expressions directly calling the research subject (for example, Patient Safety). The Figure 4 shows the visualization of significant trending terms (a cloud of words) by the frequency of their use.

Based on the word cloud, we identified three groups that are most widely discussed in the global Twitter community using the hashtag #PatientSafety, with these three groups (blocks) being “Patient,” “Practical doctors,” and “Health Care Safety Management” (Table 3 presents the three blocks together with the top trending terms that are most frequently mentioned in the #PatientSafety tweets).

**Table 3:** Trending terms that are most frequently tweeted with #PatientSafety.

	Trending terms	Frequency
“Patient” block		
1.	Patients	12,025
2.	Support	4,263
3.	Health	4,007
4.	Health care	1,626
5.	World patient safety day	836
“Practical doctors” block		
6.	Learn	8,204
7.	Improve	4,114
8.	Healthcare professionals	900
9.	Lean healthcare management	835
10.	PSNet featured research	821
11.	Health worker safety	388
“Health Care Safety Management” block		
12.	Risk	3,677
13.	Quality	3,657
14.	Harmed	3,424
15.	Medical errors	738
16.	Due to unsafe medicine practices	651

Analysis of online communications and public sentiment regarding patient safety could be of benefit for healthcare improvement. Some of the most accessible and current data acquisition sources are social media platforms such as Twitter. Analysis of such data supplements informed decisions and conclusions in medical practice.

However, limitations regarding the generalization of the results of studies of Internet platforms related to user bias should also be noted. The limitations of medical information, which can be found on social networks and other online sources, include, in some cases, insufficient quality and reliability. In addition, medical information may be unreasonable, incomplete, or unverified (25).

**Figure 5:**
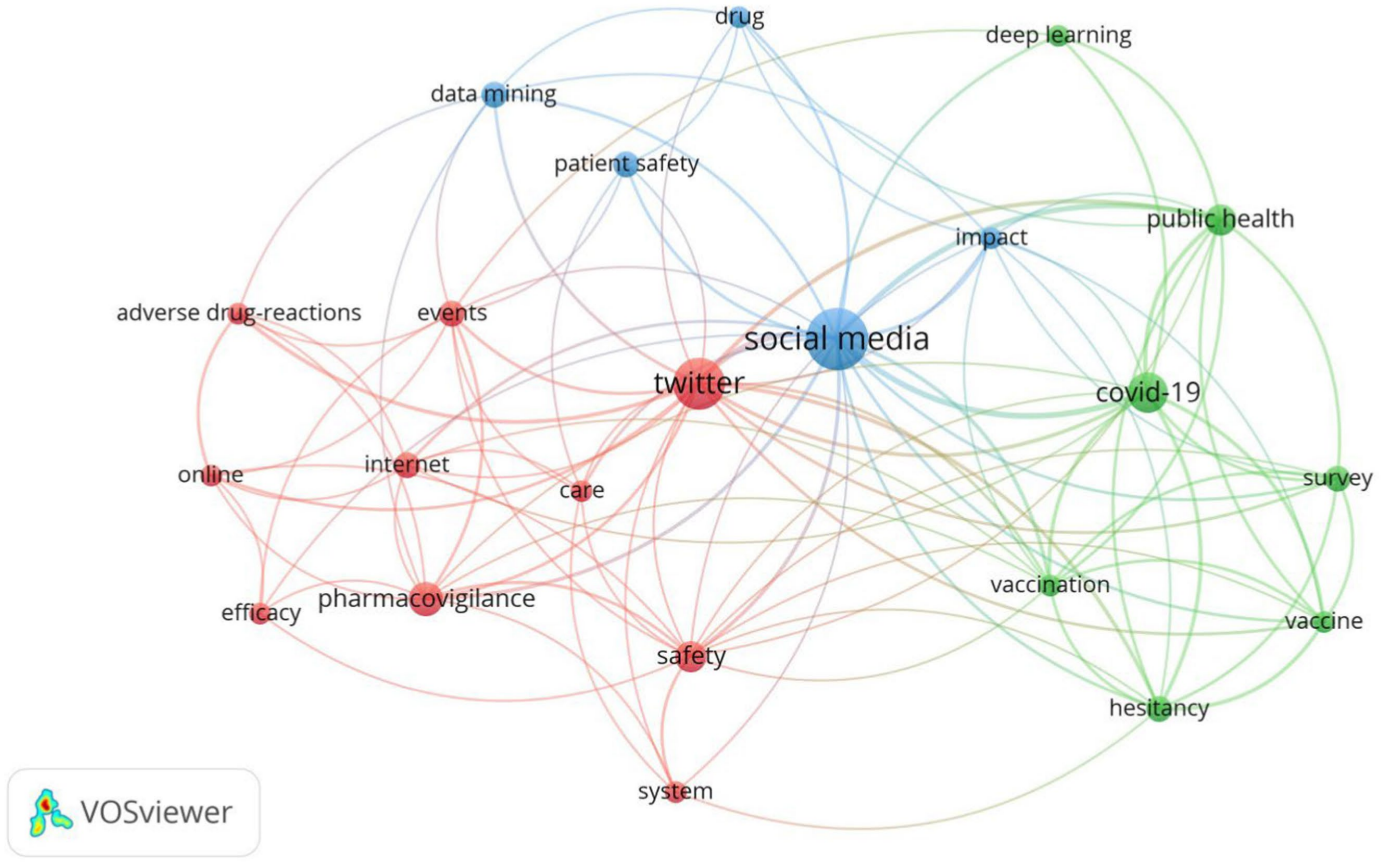
A map of the keywords of publications about patient safety discussions on Twitter in Web of Science (2000–2023).

An additional bibliometric analysis of the Web of Science database was performed using VOSviewer version 1.6.18 in the area of discussing patient safety on Twitter for the period 2019–2023. The result of the bibliometric analysis in the VOSviewer program is presented in Figure 5. The program identified three clusters similar to the Twitter analysis outcomes, which are indicated in the Figure 5 in green (Patients, ensuring their safety and COVID-19), blue (Doctors, social media opportunities), and red (Health safety management, including pharmacovigilance) colors. Circles conventionally indicate a keyword; the larger the diameter of the circle, the higher the frequency of references to the corresponding concept.

The identified main topics of discussion, according to the analysis of trending terms and bibliometric research in the Web of Science database, are presented below.

## 4. Discussion

### 4.1. Patients, ensuring their safety, and COVID-19

Real-time analysis of public attitudes related to patient safety during COVID-19, including vaccine safety and effectiveness, patient-doctor communication, and patient support by relatives and loved ones, will allow the clinician to effectively manage patient safety.

Our findings demonstrate the sustained interest of the Twitter community in the COVID-19 issue during the pandemic. It was revealed that together with #PatientSafety, the co-occurring hashtag #COVID-19 has been mentioned 11,213 times. Twitter has been used as a platform by WHO, health agencies, government organizations, hospitals, doctors, and medical journals from different countries to distribute timely information related to patient safety during COVID-19. Thereby, 21 #PatientSafety tweets were shared by WHO in the study period, and the WHO account was mentioned 7,285 times. Consequently, the population has been able to promptly receive the latest verified information provided by WHO, and the WHO has been highly regarded as a key organization with relevance to patient safety.

The analysis also confirmed that Twitter was also actively used by doctors treating patients with COVID-19 to inform the medical community and patients about their experience promptly.

Our data are consistent with results obtained by other scientists. Pangborn et al. analyzed the Twitter messages for 1 year (from March 13, 2020, to March 12, 2021) during the pandemic. The authors conclude that Twitter was used by family doctors as a platform on which they shared their experiences (26).

A block of trending patient-related terms highlights the need for active patient-doctor communication. Within the hashtag #PatientSafety, Twitter users widely discuss the issue of the safety and effectiveness of vaccination against COVID-19, and patient participation in the treatment process. Users who post tweets about the positive impact of vaccination emphasize the need to protect themselves, their families, and society; the possibility of avoiding a severe form of the disease; and doctors’ recommendations. On the other hand, there was also distrust of the vaccine, which was developed and investigated in a short time, and concern regarding possible side effects of the vaccine.

In contrast to the assumed high level of ambiguity over the safety of vaccinations against COVID-19, the findings of our prior work in real-time utilizing surveys on Twitter show that there is an increasing readiness to get vaccinated among the Twitter community participants (27). Similar results were obtained by Lyu et al. Tweets related to COVID-19 vaccines were analyzed from March 11, 2020, to January 31, 2021. Sentiment estimates showed that the sentiment was increasingly positive despite the swings. The analysis of emotions also showed that trust was the most predominant emotion, followed by expectation, fear, sadness, etc. On November 9, 2020, Pfizer stated that its vaccine was 90% effective, and the feeling of trust peaked (28). The Hoffman BL study, which includes a survey on COVID-19 vaccination and an analysis of Twitter messages, showed that encouraging the exchange of personal stories about the COVID-19 vaccine on social networks, combined with measures aimed at specific reasons for hesitancy about the COVID-19 vaccine, can be an effective means of reducing uncertainty about the COVID-19 vaccine (29).

One more problem related to patient safety during the pandemic is the diminished support of loved ones. Hriberšek et al. revealed that the Twitter community supported the notion that during the patient’s stay in the hospitals in the pandemic period, the patient’s loved ones must be involved. Both patients and their loved ones will have a better hospital experience as a result of their moral support and communication, which increases patient safety (30).

Thus, analysis of the “patient” block of trending terms revealed the following possibilities for reducing the risks of adverse events in patients: obtaining information from doctors about the advisability of vaccination and the choice of vaccines, especially for those at risk (diabetics and older adults), the symptoms, and the course of COVID-19; using the Internet of Medical Things; and the support and care of the patient from his relatives and friends.

### 4.2. Doctors, social media opportunities

The doctor’s ability to store, analyze, and use information is significantly expanding with the additional use of computer technology. The Twitter platform is widely used by healthcare professionals for additional knowledge sharing, experience exchange, and continuous professional development.

Analysis of trending terms associated with the “Practical doctors” block suggests a significant role of the human factor in ensuring patient safety. The importance of advanced training for doctors, constant communication between doctors and patients, doctors and teams, and implementing modern information technologies into medical practice (artificial intelligence, machine learning, social media platforms such as Twitter, etc.) should be noted. Meanwhile, risk factors for the healthcare quality include occupational burnout associated with excessive load and negative organizational factors.

The pandemic period provided an opportunity to understand the importance and feasibility of introducing modern digital solutions into the healthcare system. One of the trending terms in the tweets in our study was “learn.” There is no doubt that Twitter provides the opportunity for doctors to gain knowledge and information about open innovations to ensure patient safety during the COVID-19 pandemic.

In a similar context, the study of Dost et al. using the social media platforms Twitter, LinkedIn, and WhatsApp to assess the knowledge of specialist anesthesiologists and their attitude toward strategies and methods of treatment in the intensive care unit in patients with a suspected or confirmed diagnosis of COVID-19, is of interest. The latter work has revealed that the majority of doctors showed the correct attitude toward ensuring airway patency; assistant researchers with little professional experience were seen to be indecisive or inclined to make incorrect decisions (31).

In the context of education, there are also studies about the possibility of using Twitter as part of the interprofessional curriculum of the patient safety course for students and teachers (32).

There is no doubt that modern medical digital information technologies improve doctors’ working conditions and the quality of health care. Thus, it is reported that it was possible to reduce the number of erroneous medications, reduce the number of adverse events, and improve compliance with clinical recommendations when moving from paper records to electronic medical records. However, patient safety also includes digital safety. Patients aware of the effectiveness of modern information systems are concerned about the increased risk of personal information insecurity in digital databases. Blockchain technology analyzes the storage architecture of medical data and ensures that it cannot be altered or tracked (33–35).

Thus, for the second block of trending terms, the following opportunities have been identified as contributors to reduce the risks for patients and improve their safety: advanced training for doctors, constant communication between doctors and teams, international sharing of best practices by doctors, and the implementation of modern information technologies into medical practice [artificial intelligence (machine learning), social media communications etc.].

### 4.3. Health safety management, including pharmacovigilance

The modern approach to drug and vaccine development is based on a deep understanding of the nature of diseases and use of the latest achievements in pharmacy, and it combines the experience of classical medicine and modern technologies. Twitter might additionally help to identify public willingness for vaccination and has been utilized to detect adverse events of medicines and vaccines, thereby strengthening modern pharmacovigilance systems that rely on spontaneous reporting and health observations.

The third block of trending terms of the #PatientSafety tweets, “Health Care Safety Management,” relates to tools to combat adverse events in medicine.

The pharmacovigilance system plays an important role in ensuring the safety and effectiveness of medicine and patient therapy. The use of any medicine is always associated with the risk of adverse events. Preventing adverse events with the use of medicines is more rational than taking measures to eliminate them, which determines the need for risk management. It should be noted that Twitter has the potential to serve as additional source to identify adverse events associated with the use of medicines, including vaccines.

An analysis of trending terms and co-occurring hashtags revealed that Twitter participants actively participated in conversations related to the risk of adverse events from both vaccines against COVID-19 and other medicines. These findings are in line with the results of other authors.

Thus, Lian et al. analysed tweets (from December 1, 2020, to August 1, 2021) containing personal experience with COVID-19 vaccinations, as well as information about the dose, type of vaccine (Pfizer, Moderna, and Johnson & Johnson), and symptoms. It has been established that the four most populous US states (California, Texas, Florida, and New York) had the most discussions about adverse events on Twitter. The frequency of Twitter discussions of adverse events coincided with the course of the COVID-19 vaccination. Touch tenderness, fatigue, and headaches were the three most common adverse effects of all three COVID-19 vaccines in the United States. The authors conclude that it is possible to use social media data to monitor adverse events (36).

Bennett et al. based on the analysis of adverse hematological events associated with vaccines using the Twitter platform, note the need to improve pharmacovigilance approaches (37). Despite the fact that social networks contain information noise and do not have the ability to check the accuracy of patients’ messages about possible adverse events, they also have advantages due to their coverage and depth.

Thus, the accelerated approval of vaccines to combat the COVID-19 pandemic emphasized the need to quickly obtain data on their safety in the post-marketing period. A possible additional source of data is Twitter.

The interest of Twitter users using the hashtag #PatientSafety in the problem of adverse events to vaccines and other medicines has also been identified. The main flow of relevant publications is devoted to using of medicines from different pharmacotherapeutic groups. These findings fit well with the results of other authors.

Li et al. noted that the modern pharmacovigilance system for medicine safety relies on spontaneous reporting systems and data from health observations (38). However, the detection of adverse drug events may occur with delay and a lack of geographic diversity. The researchers extracted potential adverse event reports from Twitter, as they did from the US FDA (The United States Food and Drug Administration) Adverse Event Reporting System, and then integrated those signals. The authors conclude that the accuracy of signal detection using social networks can be improved by combining Twitter signals with signals from the spontaneous message system. However, further research is needed to use an integrated system that includes Twitter.

The following previous works have explored the use of Twitter as an additional element of pharmacovigilance.

Golder et al. evaluated the consistency of data on the side events of statins from Twitter social media compared to other sources. It was revealed that most adverse events showed a high level of agreement between Twitter and regulatory data. While being a complex approach, pooling of data from multiple sources can provide a broader safety profile for any drug (39). Similar results were obtained by Smith et al. in analyzing tweets mentioning adalimumab in relation to adverse events (40).

Patel et al. assessed glucocorticoid-related adverse events using Twitter and spontaneous reports of adverse events to the national drug regulatory authority. The authors conclude that pharmacovigilance using Twitter data could be a valuable additional source of information on drug safety (41).

Thus, studies demonstrate consistency between adverse event data from Twitter and regulatory bodies, emphasizing the value of multi-source data in creating a comprehensive drug safety profile and, as a consequence, patient safety.

On the other hand, social networks serve as a platform for disseminating information about the adverse events of drugs. When safety concerns involving pharmaceutical drugs that have received FDA approval arise, the FDA releases drug safety communications to patients, healthcare providers, and the general public. Social media is used to spread these safety messages and guarantee their widespread adoption. In this context, Sinha et al. evaluated the spread of two posts on social media (Twitter and Facebook) for sleeping pills zolpidem. The authors conclude that social networks have opportunities to distribute drug safety communication messages for preventing adverse events for patients (42).

Another problem that Twitter users are discussing with the hashtags #PatientSafety (and in addition often with #COVID-19) is the “off-label” use of medical products. “Off-label” use of a medical product means its use in situations where such a product is intentionally used for medical purposes but does not correspond to the information approved by regulatory authorities about it.

Among the major healthcare problems in the first waves of the pandemic was the lack of vaccines and drugs for the treatment and prevention of COVID-19. This led to the use of many off-label drugs (hydroxychloroquine, azithromycin, etc.) for treating patients with COVID-19 (43). In this regard, pharmacovigilance information shared on social networks, including Twitter, also plays an important role in identifying risks.

During the COVID-19 pandemic, four drugs received a lot of public attention: hydroxychloroquine, ivermectin, the latter both representing drug therapies with anecdotal evidence, and molnupiravir and remdesivir, both of which represents FDA-approved treatment options for eligible patients. Hua et al. looked into 609,189 tweets from the US between January 29, 2020, and November 30, 2021. The study demonstrated how social network users view and react differently to drug use that is not for a purpose for which the FDA approved them and that occurs at various COVID-19 stages (44). This suggests that in order to promote safe medication use, health systems, regulators, and legislators should establish targeted ways to monitor and lower disinformation.

Thus, analysis of all posts, including on Twitter and other social networks, including both positive and negative research results, is necessary to present a complete picture of the safety profile of drugs as perceived by users, in the context of patient safety. Acquiring of such information may prevent adverse events in patients later on. It is worth noting that such information might benefit the joint work of drug manufacturers and regulatory authorities, to provide more complete protection for the patient from adverse reactions and prevent the development of serious adverse events.

To better comprehend the public mood and concerns surrounding adverse drug and vaccine responses, it should be mentioned that medical organizations, pharmaceutical corporations, and regulatory agencies may also use publicly available information offered by the public on social media.

According to the WHO, medication errors are among the most important healthcare problems. This matter is also actively discussed by Twitter users and scientists in scientific publications. Among the trending terms in our study, “medical errors” should also be noted. Medical errors can have serious consequences for the patient.

A retrospective study has found that the majority of medication errors were caused by dosing errors and errors in drug frequency, mostly attributed to physicians among hospitalized patients with COVID-19 in Saudi Arabia. The most frequent drug categories implicated in medication errors and adverse events, respectively, were antibiotics (32%), and antineoplastics (25%) (45).

Makary et al. reported that adverse events are the third leading cause of death in the US population (46). Although the results of this study have been criticized by scientists (47), the relevance of the problem is not in doubt. Reason et al. in their work suggest using a systematic approach in managing adverse events (48).

Additional use of Twitter data analysis might aid the establishment of a systematic approach to managing patient safety. Publicly available information on social media is useful for healthcare organizations and regulatory agencies to understand public sentiment on drug safety and monitor medication errors.

The analysis performed allowed us to formulate generalized approaches to strategies for reducing the risks of adverse events in patients, as follows.

### 4.4. Summarized approaches for strategies to reduce the risks of adverse events in patients

The current study reveals the discussions and attitudes of Twitter users toward patient safety during the COVID-19 pandemic. Such kind of analysis might yield real-time understanding of public sentiment about the question under investigation, thereby contributing to understanding the evolving situation. Thus, this type of research gets beyond the drawbacks of the conventional social science technique, which is based on small-scale, time-consuming, retrospective, and delayed interviews and surveys, and provides a valuable additional source of information.

**Figure 6:**
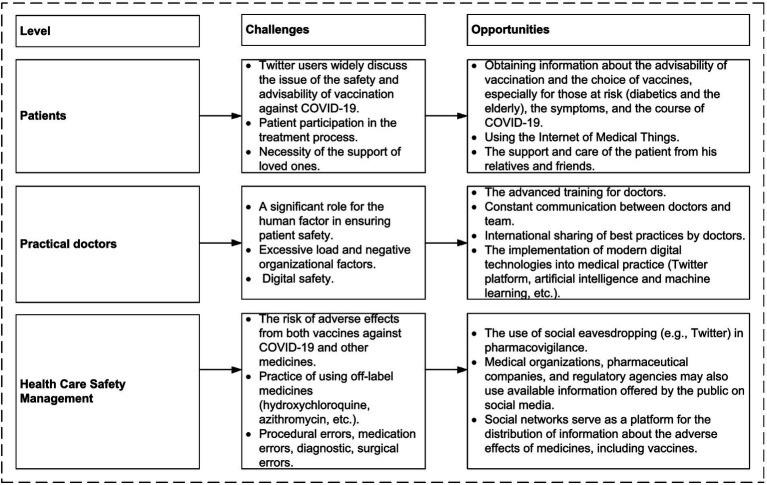
Summarized approaches to reduce the risks of adverse events reflected in the #PatientSafety discussions on Twitter.

Summarized approaches for strategies to reduce the risks of adverse events reflected in the #PatientSafety discussions on Twitter are shown in Figure 6.

It should be noted that along with the benefits of additional use of the Twitter platform for managing patient safety, there are a number of limitations. Health misinformation was widespread online during the COVID-19 pandemic (49). The WHO declared it an “infodemic,” reflecting an overabundance of accurate and inaccurate information that makes it difficult for people to identify reliable information sources. The spread of misinformation contributes to delayed treatment, patient anxiety, and harm to health. In this context, Skafle et al. summarized the results of 19 studies of social media misinformation about COVID-19 vaccines and their implications (50). These studies show that social media misinformation has a negative impact on vaccine trust and use. The authors conclude that further research is needed for the benefit of public health. In connection with this phenomenon, WHO has developed recommendations in 2020 to address misinformation (51). It is suggested that social media companies be involved in the dissemination of credible information. It is planned that social media platforms will implement mechanisms to warn users about the presence of misinformation in the content, including based on machine learning.

The WHO policy brief (2022) (52) offers a range of policy recommendations for all stakeholders: increasing digital literacy, arguing the need to combat the infodemic, providing secure online platforms, establishing multi-stakeholder networks to combat the infodemic, improving risk communication, and implementing ongoing monitoring of harmful and deceptive content on the Internet.

Future research may help to develop superior strategies to overcome the above-mentioned infodemic obstacles and improve patient safety management.

## 5. Conclusion

The use of digital technologies together with the latest advances in medicine is an important approach in improving health conditions, ensuring patient safety, and extending life. The following areas of possible use of Twitter as a valuable additional source of information were identified in the present work: ensuring patient safety during COVID-19; using Twitter by physicians to share additional knowledge, experience, and continuing professional development; Twitter as an additional element of pharmacovigilance, an additional monitor of medical errors in taking drugs.

Thus, the performed Twitter analysis established that the components of the patient safety process reflected in the online discussions are: active communication between the patient, the doctor, and loved ones; the support and care of the patient from loved ones; advanced training of doctors; team communication; international sharing of best practices by doctors; implementation of modern digital technologies in medical practice; a system approach to preventing adverse events, including pharmacovigilance, in particular using feedbacks collected through the Twitter platform; and the timely analysis and prevention of the causes of medical errors. In order to prevent misinformation on social networks, including Twitter, implementation of the WHO recommendations to warn users about the presence of misinformation in the content could be a promising approach, including applications based on machine learning.

Summarized approaches for patient safety promotion can be used in other pandemics and for patient safety management in clinical practice.

The study has several limitations. Firstly, we only analyzed one hashtag used in Twitter discussions. Secondly, Twitter users are not representative of the entire population, and the collected data only indicate the opinions and reactions of online users possessing Twitter accounts. In this context, it is of interest to study data from other Internet platforms (for example, Facebook, Instagram, YouTube, etc.) and analyze their correlation. It is also promising to focus on discussions shared in different languages to analyze the reactions of the populations in specific countries. In future studies, it would be promising to include German, French, and other languages for analysis in order to obtain a global perspective. Notably, studies of online discussions are time-sensitive (e.g., in the present study the tweets were collected in a specified time period). In summary, with the careful consideration of existing limitations, Twitter data overall represent a valuable source of information for real-time gain of knowledge based on user-generated content related to patient safety.

## Data availability statement

The original contributions presented in the study are included in the article/supplementary material, further inquiries can be directed to the corresponding authors.

## Author contributions

OL: Conceptualization, Investigation, Methodology, Writing – original draft. FM: Writing – review & editing. MM: Writing – review & editing. BZ-K: Writing – review & editing. CT: Writing – review & editing. BS: Writing – review & editing. JS: Writing – review & editing. AA: Conceptualization, Data curation, Formal analysis, Investigation, Methodology, Project administration, Software, Supervision, Writing – review & editing. HW: Conceptualization, Investigation, Writing – review & editing.

## Footnotes

^1^ https://help.symplur.com/en/articles/103684-healthcare-stakeholder-segmentation
